# Exploring the Antibacterial Activity of *Ipomoea staphylina* Extracts Against *H. pylori*: A Pharmacognostic Investigation of Whole Plant and Matured Stem with Emphasis on Quercetin Isolation

**DOI:** 10.21315/tlsr2024.35.3.10

**Published:** 2024-10-07

**Authors:** Lakshmanan Narayanan, SR Suseem

**Affiliations:** Department of Chemistry, School of Advanced Sciences, Vellore Institute of Technology, Vellore – 632014, Tamil Nadu, India

**Keywords:** Pharmacognostical Evaluations, Phytochemical Screening, Fluorescence Analysis, HPTLC Analysis, Quercetin

## Abstract

*Ipomoea staphylina* Rome & Schult, entrenched in ethnomedicinal practices, is recognised for its efficacy in treating stomach disorders. Traditionally used in Dharmapuri, Tamil Nadu for stomach ulcers, its matured stem bark latex is therapeutically relevant, especially for *Helicobacter pylori (H. pylori)* infections. This prompts scientific exploration into its antibacterial properties. The research validates the antibacterial efficacy of *I. staphylina* extracts against *H. pylori*, scrutinising the whole plant and matured stem through a comparative pharmacognostic analysis. Utilising herbal standardisation techniques, we confirm the heightened purity of the powder. Antimicrobial assessments show exceptional efficacy of DME (dried Ethanolic extract of *I. staphylina*) and HLS (hydro alcoholic extract of *I. staphylina*) extracts. Quercetin isolation by using advanced instrumentation (Nuclear magnetic resonance [NMR], High resolution mass spectrometry [HR-MS], High-performance thin-layer chromatography [HPTLC], Fourier transform infrared spectroscopy [FTIR]) ensures precise compound identification. This methodology guarantees an exhaustive analysis, confirming purity and identifying bioactive components. Standardisation underscores the elevated purity of *I. staphylina*, with phytochemical screening revealing a predominant presence of phenolics and flavonoids. Antibacterial investigations highlight significant activity, particularly with DME and HLS extracts. These findings substantiate *I. staphylina’*s medicinal significance, especially its matured stem latex, as a promising treatment for *H. pylori*-induced stomach ulcers, affirming traditional use by Dharmapuri villagers.

**Highlights**
- Research confirms the antibacterial efficacy of *Ipomoea staphylina* extracts against *H. pylori*. Study involves pharmacognostic analysis of the whole plant and matured stem.
- Phytochemical findings: High presence of phenolics and flavonoids in the extracts. Advanced instrumentation ensures precise identification of quercetin.
- Medicinal significance and antimicrobial efficacy: DME and HLS extracts show exceptional antibacterial activity. Findings support the use of *Ipomoea staphylina’s* matured stem latex as a treatment for *H. pylori*-induced stomach ulcers.

## INTRODUCTION

Herbal plants have been utilised for centuries in the treatment of various diseases (Balasubramaniam *et al*. 2020; Süntar 2020). Medicinal plants and their isolated compounds have (Maqbool *et al*. 2019) played a significant role in both conventional and alternative medicine for thousands of years (Maqbool *et al*. 2019; Süntar 2020). For instance, Cinchona bark has been traditionally used in South America for the treatment of malaria (Tolkushin *et al*. 2020). Notably, many well-known medications, including Curcumin, Digitoxin, Caffeine and Atropine, are derived from plants (Maqbool *et al*. 2019). The World Health Organisation (WHO) estimates that around 21,000 plant species have been utilised globally for medicinal purposes (Sundaramoorthy *et al*. 2021). Herbal medicines form the cornerstone of various alternative treatments that have gained popularity in recent years (Maqbool *et al*. 2019; Süntar 2020). Moreover, plant-based medications have a profound impact on drug development and design (Gomathi *et al*. 2012). The increased utilisation of herbal-based products can be attributed to their efficacy, affordability, reduced toxicity and eco-friendliness (Zhou *et al*. 2007). In fact, approximately 60% to 75% of cancer and infectious disease medications are derived from natural sources (Newman *et al*. 2000; Song *et al*. 2014). Given their ability to interact with proteins, herbs are considered valuable in drug design, contributing to the creation of novel medications (Newman *et al*. 2000; Song *et al*. 2014).

*Ipomoea staphylina* Rome & Schult, a plant deeply entrenched in ethnomedicinal practices, boasts a spectrum of biological properties particularly recognised for its efficacy in addressing stomach disorders (Dias *et al*. 2012; Narra & Kandavara 2014), the matured stem bark latex of this plant has been traditionally employed by communities in Dharmapuri, Tamil Nadu, for the treatment of stomach ulcers. The therapeutic relevance is noteworthy, especially considering that stomach ulcers are often attributed to *Helicobacter pylori (H. pylori)* bacterial infections (Majumdar & Looi 2024). This traditional application underscores the potential of *I. staphylina* as a valuable resource in managing gastric ailments, providing a rationale for scientific exploration into its antibacterial properties against *H. pylori*. Most commonly in human *H. pylori* is the main reason for the stomach ulcer (Youssefi *et al*. 2021). *H. pylori* is a gram-positive bacterium that infects the stomach (Kishikawa *et al*. 2020; Youssefi *et al*. 2021). *H. pylori* infection typically occurs during childhood (Kishikawa *et al*. 2020; Lee 2019; Majumdar & Bebb 2019). The bacteria can be transmitted through contaminated water, food or contact with the saliva, vomit or faeces of an infected person. *H. pylori* colonise the stomach lining, causing inflammation and weakening the protective mucous layer (Alexander *et al*. 2021). This can lead to the development of peptic ulcers, including gastric ulcers (in the stomach) and duodenal ulcers (in the upper part of the small intestine (Alexander *et al*. 2021; Kishikawa *et al*. 2020; Lee 2019; Majumdar & Bebb 2019; Youssefi *et al*. 2021). We can control stomach ulcers by controlling this bacterial growth. The principal objective of this research is to assess the antibacterial efficacy of *I. staphylina* against *H. pylori*, thereby substantiating its traditional use for anti-ulcer purposes in the Dharmapuri village. Additionally, the study aims to elucidate the traditional practices associated with *I. staphylina* in treating stomach ulcers within the Dharmapuri village community.

*I. staphylina* is an important climber known for its use in the treatment of respiratory problems, as an anthelmintic, purgative, for bronchitis, and for stomach ailments (Narra & Kandavara 2014; Raghavendra 2013; Ramesh, Deepa, *et al*. 2022; Ramesh, Rajeshkumar, *et al*. 2022). This plant exhibits a range of pharmacological effects, including antimicrobial, anti-inflammatory, antioxidant, anti-diabetic and anti-mutagenic properties (Raghavendra 2013). We have done the quantitative phytochemical screening of the plant extract, employing standardised methodologies (Krishnaveni *et al*. 1984; Soni & Sosa 2013; Yilmaz 2020). This analysis provided valuable insights into the composition and concentration of secondary metabolites inherent to the extract. Concurrently, the assessment of antioxidant potential was pursued using the DPPH (2,2-diphenyl-1-picrylhydrazyl) assay, recognised for its efficacy in gauging radical scavenging activity (Isildak *et al*. 2022; Sahidin *et al*. 2022; Sihag *et al*. 2022). The resulting data showcased a commendable percentage of radical scavenging activity, underscoring the extract’s promising antioxidant capabilities. These findings collectively contribute to a deeper understanding of the extract’s chemical composition and potential applications in various fields. To confirm the presence of secondary metabolites in the plant extract, crude drugs underwent High Performance Thin Layer Chromatography (HPTLC) analysis with standard drugs (Saxena *et al*. 2021). HPTLC was employed to identify and authenticate the phytochemicals and markers present in the *I. staphylina* plant extract (Gunjal Sanket & Dighe 2022; Mir *et al*. 2020). Through comprehensive antibacterial evaluations, the research endeavours to contribute empirical evidence supporting the plant’s potential therapeutic applications, aligning traditional knowledge with contemporary scientific scrutiny in traditional medicine.

### Ethnopharmacology

*I. staphylina*, a plant of significant therapeutic importance, has been utilised for the treatment of various disorders, including purgation, stomach disorders, pain, rheumatism and inflammation (Firdous & Koneri 2014). In the region of Dharmapuri, the matured stem latex of *I. staphylina* has been traditionally employed to address stomach ulcers. Similarly, the residents of Gingee Hills have utilised leaf latex for the treatment of foot cracks (Muralidharan & Narasimhan 2013). Notably, the roots of *I. staphylina* have been used as an antidote for snake bites by the Irula and Palliyar tribes (Sarvalingam *et al*. 2014). Additionally, villagers in Karandamalai have administered a leaf decoction to alleviate stomach problems (Kottaimuthu 2008). The Chenchus tribes have employed leaf extract as a treatment for piles (Kumar T D & Pullaiah 1999). These traditional uses underscore the diverse therapeutic potential of *I. staphylina* in various cultural settings.

## MATERIALS AND METHOD

### Chemical and Reagents

Solvents including methanol, ethanol, ethyl acetate and petroleum ether were procured from SD Fine Chemicals (India). Additionally, CHCl_3_, NaOH, KOH, HCl, H_2_SO_4_, HNO_3_, Na_2_CO_3_, acetic acid and DPPH were obtained from Avra Chemicals (India). Reagents required for qualitative phytochemical testing, such as neutral FeCl_3_, alkaline reagent, Mayers solution, Benedict’s reagent, Salkowski reagent, Fehling’s solution and Folin-Ciocalteu reagent, were sourced from HiMedia (India).

### Plant Collection

Plant parts were collected from the Dharmapuri region (Tamil Nadu, Latitudes N11°47′ and 12°33′ and Longitudes E77°02′ and 78°40′) during the winter season of December 2020. The soil in which the plant was grown is characterised as clay. To ensure accurate identification, the plant specimen has been authenticated by the Botanical Survey of India, Southern Region Coimbatore (BSI/SRC/5/23/2022/Tech/429).

### Plant Extraction

This research investigation focuses on two specific components of *I. staphylina*, specifically the stem and leaves and matured stem, as targets for study. Fresh plant parts were subjected to extraction using two distinct methods: hot continuous percolation (Soxhlet) and immersion. Additionally, the Soxhlet method was followed for the extraction of bioactive compounds from the dried parts (dried under sunshade) of *I. staphylina* (Bag & Mumtaz 2013; López-Bascón & De Castro 2020). These chosen extraction techniques were carefully selected to ensure optimal recovery of valuable constituents from the plant material, facilitating subsequent analysis and characterisation.

### Fresh Part Extraction

**Figure 1:**
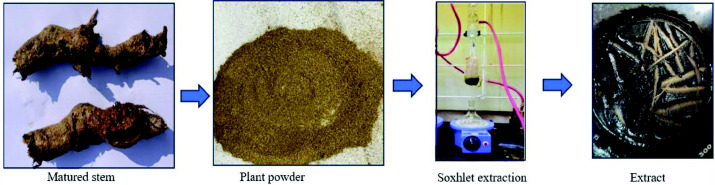
Fresh part extraction matured stem of *I. staphylina*.

Fig. 1 shows the fresh plant part extraction of *I. staphylina*. Fresh plant parts were meticulously washed with distilled water to remove any impurities, followed by pulverisation using an electronic blender. The resulting blend was subjected to extraction using two distinct methods: Soxhlet and immersion (Bag & Mumtaz 2013; López-Bascón & De Castro 2020). In the Soxhlet method, solvents such as petroleum ether, ethanol and hydroalcohol (a mixture of 80% methanol and 20% water) were used. Conversely, for the immersion method, distilled water served as the solvent. Following the extraction process, the solvents were evaporated using a Rotavapor R-100 apparatus. The resulting dried crude extract was carefully collected in an airtight container and stored under refrigeration conditions. These standardised procedures ensure the preservation and stability of the extracted compounds for further analysis and experimentation.

### Dry Part Extraction

**Figure 2:**
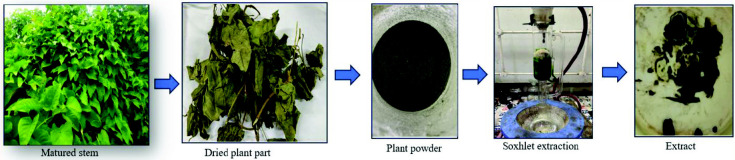
Dried part extraction stem and leaf of *I. staphylina*.

Fig. 2 shows the extraction process of dried plant parts of *I. staphylina*. The *staphylina* plant parts (matured stem and stem and leaf) were carefully dried under shade at room temperature for 10 days, ensuring the preservation of their chemical constituents. Subsequently, the dried parts were finely pulverised using an electronic blender, resulting in a homogeneous powder. The extraction process was conducted using the Soxhlet method, employing solvents such as petroleum ether, ethanol and hydroalcohol (a mixture comprising 80% methanol and 20% water) (Bag & Mumtaz 2013; López-Bascón & De Castro 2020). This extraction method facilitated the efficient extraction of bioactive compounds from the plant material. Following the extraction, the solvents were evaporated using a Rotavapor R-100 apparatus. The resulting dried crude extract was carefully collected and stored in an airtight container, ensuring protection from moisture and other degrading factors. To maintain the stability and integrity of the extracted compounds, the container was stored in a refrigerator, providing optimal storage conditions (Jeyadevi *et al*. 2019). These meticulous steps in sample preparation and storage guarantee the quality and usability of the extracted material for further scientific analysis and investigation.

### Physicochemical Standardisation

**Figure 3:**
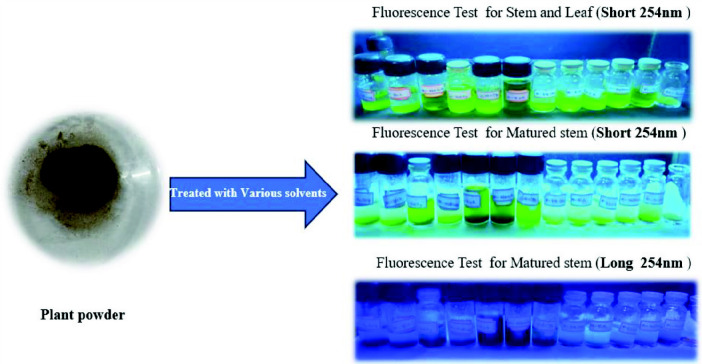
Fluorescence analysis of *I. staphylina*.

Fig. 3 shows the florescence test of *I. staphylina* plant powder. Before commencing any research work on a specific herb, it is essential to conduct a comprehensive assessment of its physicochemical parameters (Snehalatha & Rasmi 2021). In order to standardise the herbal powder, various pharmacognostic characteristics were evaluated, including ash value, loss on drying, acid-insoluble ash, water-soluble ash and fluorescence analysis (Khanal *et al*. 2018; Khandelwal 2008; Snehalatha & Rasmi 2021). These physicochemical standardisations were performed according to established protocols and guidelines. By examining these pharmacognostic characteristics, it becomes possible to identify potential adulterants and impurities present in the herbal drug, ensuring the purity and quality of the material under investigation. These evaluations serve as crucial preliminary steps in the research process, providing valuable insights into the composition and integrity of the herb being studied.

## EXPERIMENTAL SECTION

### Pharmacognostic Evaluations

Prior to initiating any research, the standardisation of herbal drugs is imperative (Khandelwal 2008). In the case of *I. staphylina* herbal powder, pharmacognostic evaluations were conducted to ensure its quality and authenticity. These evaluations encompassed essential parameters, including loss on drying, ash value, water-soluble ash and acid-insoluble ash, the results of which are presented in Table 1 (Khanal *et al*. 2018; Khandelwal 2008). These rigorous tests serve the purpose of safeguarding against potential adulterants and maintaining the integrity of the herbal drug. Furthermore, fluorescence analysis was employed as a critical parameter to assess the purity and quality of the powdered drug material. These comprehensive assessments collectively contribute to the standardisation process, guaranteeing the reliability and effectiveness of the herbal drug under investigation.

### Fluorescence Analysis

Fluorescence analysis of the powdered drugs was conducted following a standardised procedure (Das *et al*. 2021; Khanal *et al*. 2018; Khandelwal 2008). To perform the analysis, various solvents including water, methanol, ethanol, ethyl acetate, petroleum ether, chloroform, 10% sodium hydroxide, 10% potassium hydroxide, hydrochloric acid (4N), sulphuric acid (4N), nitric acid (4N) and acetic acid (4N) were individually mixed with the desired herb powder. After allowing sufficient time for interaction, the fluorescence of the plant powder was examined under both ultraviolet (UV) and visible light. The obtained fluorescence patterns provide valuable insights into the unique fluorescent characteristics of the matured stem, stem and leaf powders, as depicted in Tables 2 and 3, respectively. This fluorescence analysis serves as an important tool for the characterisation and assessment of the quality of the herbal drug, contributing to its standardisation and reliable utilisation in further research endeavours (Amir *et al*. 2019; Bashir *et al*. 2019; Jain *et al*. 2021; Misra *et al*. 2018; Ved *et al*. 2022).

### Qualitative Phytochemical Screening

The extracted crude samples underwent a qualitative phytochemical investigation to identify the presence of various phytoconstituents, including alkaloids, tannins, flavonoids, phenols, saponins, proteins, sterols, terpenoids, carbohydrates, and fats and oils, utilising established standard methods (Amir *et al*. 2019; Banerjee & Firdous 2020; Bashir *et al*. 2019; Das *et al*. 2021; Harborne 1998; Jain *et al*. 2021; Khanal *et al*. 2018; Khandelwal 2008; Misra *et al*. 2018; Tiwari & Patel 2012; Ved *et al*. 2022). The results of the preliminary phytochemical screening of the freshly dried parts of *I. staphylina* are presented in Tables 4 and 5.

### Quantitative Phytochemical Screening

The quantitative phytochemical screening was done by following standard procedure and using spectrophotometer (Isildak *et al*. 2022; Krishnaveni *et al*. 1984; Soni & Sosa 2013; Yilmaz 2020).

### Estimation of Phenols

Phenols were quantified using the FC (Folin-Ciocalteu reagent) technique, slightly modified in accordance with the procedures outlined in the reference. Initially, a 100 μL aliquot was drawn from a 10 mg/mL stock solution, with subsequent adjustment of the volume in each test tube to 3.0 mL through the addition of distilled water. The experimental protocol encompassed the sequential addition of 0.5 mL of Folin-Ciocalteau reagent and 2 mL of 20% Na_2_CO_3_ solution into the tubes. The resulting mixture was subjected to precise boiling within a water bath for a duration of 1 min. After this, the tubes underwent a cooling phase, following which absorbance measurements were taken at a wavelength of 650 nm, employing a spectrophotometer and utilising a reagent blank as a reference standard. Notably, gallic acid functioned as the standard substance throughout this investigation. To prepare the gallic acid solution, 1 mg of the compound was dissolved in 1 mL of ethanol. Subsequently, aliquots of this solution, measuring 50 μL, 100 μL, 150 μL, 200 μL and 250 μL, were meticulously extracted for subsequent analytical procedures.

### Estimation of Flavonoids

Total flavonoid content was determined using the aluminium chloride colorimetric assay. A reaction mixture containing 1 mg of extract and 1 mL of distilled water was prepared. Sequential addition of 0.30 mL of 5% sodium nitrite, followed by 0.3 mL of 10% aluminium chloride after 5 min, was carried out. After another 5 min, 2 mL of 1M sodium hydroxide was added and the solution was diluted to 10 mL with distilled water. Reference standard solutions of Quercetin (20 μg/mL–100 μg/mL) were prepared similarly. Absorbance was measured at 510 nm against a reagent blank using a UV/Visible spectrophotometer. Total flavonoid content was expressed as μg QE/mg of extract.

### Estimation of Tannins

The determination of tannin content in the sample was executed utilising the Folin-Ciocalteu method. This approach relies on colorimetric assessment, wherein the formation of a blue colour arises from the reduction of phosphotungstomolybdic acid by tannin-like compounds under alkaline conditions. To proceed, a solution containing 1 mg of extract or standard tannic acid (ranging from 50 μL to 250 μL) was prepared, achieving a final volume of 7.5 mL with distilled water. Subsequently, 0.5 mL of Folin-Ciocalteu reagent and 1 mL of 35% sodium carbonate solution were introduced. Further volume adjustments were made to reach a total of 10 mL using distilled water, following which absorbance measurements were taken at 700 nm. This method facilitated the precise quantification of tannin content in the sample

### Estimation of Carbohydrates

Total carbohydrate quantification employed the phenol sulphuric acid method with spectrophotometric analysis (Krishnaveni *et al*. 1984). Each sample (100 μL) and standard underwent a controlled three-hour incubation in a water bath with 5 mL of 2.5 N HCl, followed by neutralisation with solid sodium carbonate. After volume adjustment (100 mL) and centrifugation, a series of test tubes received working standard volumes (50 μL–250 μL), phenol solution (1 mL) and 96% H_2_SO_4_ (5 mL). Following agitation and a 20-min water bath incubation, absorbance at 490 nm was measured. Carbohydrate content was quantified using glucose as the standard reference.

### Antioxidant Activity

The investigation into the scavenging capacity of free radicals within the crude extracts was executed utilising a spectrophotometric methodology, with minor adaptations in accordance with the protocol stipulated by the reference (Isildak *et al*. 2022; Sahidin *et al*. 2022; Sihag *et al*. 2022). The samples and the established standard underwent meticulous preparation at varying concentrations of 50 mg/mL, 100 mg/mL, 150 mg/mL, 200 mg/mL and 250 mg/mL, all dissolved in a methanolic medium. A solution of 0.2 mmol/L concentration of DPPH was meticulously diluted in 50 mL of methanol, subsequently subjected to an incubation period of 30 min under controlled room temperature conditions, following which the absorbance was quantified at a specific wavelength of 517 nm. For precise quantification, the Microsoft Excel platform was employed to compute the percentage values indicative of the Radical Scavenging Activity (RSA), employing the formula mentioned below. Notably, Rutin was utilised as the reference standard throughout the experimental endeavour.

The RSA% inhibition was mathematically defined by:


(1) % inhibition=[(Control Abs)-(Sample Abs)/(Control Abs)]×100

### HPTLC Analysis

#### Sample preparation

All extracts were weighed at a concentration of 5 mg/mL and dissolved in specific solvents. The resulting solutions were subjected to sonication for 5 min and then filtered using Whatman filter paper to remove any particulate matter. Precoated TLC (thin-layer chromatography) aluminium sheets with silica gel 60 F 254 (Merck) were utilised for the analysis. The samples were applied to the TLC plates using a Linomat 5 sample applicator, ensuring a band length of 5 mm and a speed of 150 nL/sec. This meticulous procedure ensures precise and uniform application of the extracts onto the TLC plates, enabling accurate separation and identification of the individual components present in the extracts (Kumar A *et al*. 2010; Swaroop *et al*. 2005; Tiwari & Patel 2012).

#### Mobile phase

Following the elution process optimising with various solvent combinations, a suitable mobile phase was established by employing a mixture of toluene, ethyl acetate, formic acid and glacial acetic acid in a ratio of (2:6:1:1). For the preparation of samples and standards, 1 mg of the compound was dissolved in 10 mL of the respective solvent. The application of these solutions was carefully performed by marking spots on precoated TLC aluminum sheets coated with silica gel 60 F 254 (Merck). These highly specific and controlled procedures facilitate the precise separation and analysis of the compounds present in the samples, ensuring accurate results and reliable identification of individual components.

#### Chromatogram and scanning

Chromatogram development was conducted using a twin trough glass chamber for a duration of 20 min, with a distance of 80 mm, employing a mobile phase consisting of toluene, ethyl acetate, formic acid and glacial acetic acid in a ratio of (2:6:1:1). Following the development process, the air-dried TLC plates were subjected to examination under UV light. Subsequently, scanning of the plates was performed using a CAMAG HPTLC Densitometer (Scanner) equipped with Deuterium light and CAMAG winCATS software, using wavelengths of 254 nm and 366 nm. The obtained results from the HPTLC analysis are presented in Figs. 2 and 3, providing valuable insights into the separation and detection of the analysed compounds.

#### Column chromatography

To isolate quercetin, the ethanolic extract of *Ipomoea staphylina* (ESL) was chosen based on the quantity and the results obtained from high-performance thin-layer chromatography (HPTLC). For the precise analysis of 10 g of the plant extract, column chromatography was performed using a stationary phase of silica gel (60–120 mesh) (Davies & Johnson 2007; Johnston *et al*. 2013; Reid & Sarker 2012). The mobile phase used in column chromatography was similar to the one used in HPTLC (composed of a mixture of ethyl acetate, formic acid, glacial acetic acid and water in a ratio of 20:2:2:1). The yellow-coloured spot, corresponding to quercetin, was isolated and verified using thin-layer chromatography (TLC) alongside a standard sample, confirming their identical retention factor (Rf) values. The resulting product was then dried and prepared for further analysis and characterisation (Meena & Patni 2008; Sambandam *et al*. 2016; Zahoor *et al*. 2018).

### Characterisation

#### FTIR and NMR spectrum analysis

The isolated compound was subjected to Fourier-transform infrared spectroscopy (FTIR) analysis to verify the presence of specific functional groups and obtain information about its molecular structure. The FTIR results provided confirmation of the functional groups present in quercetin (Sambandam *et al*. 2016).

Additionally, the isolated compounds underwent Nuclear Magnetic Resonance (NMR) spectroscopy to determine the precise positioning of the proton and carbon binding sites (Gülşen *et al*. 2007). The analysis was performed using a Bruker 400 Mz instrument, and the obtained results were processed using Topspin software version 3.6.2. The NMR spectra, depicting the chemical shifts and peak patterns, are presented in Figs. 5 and 6, allowing for a detailed characterisation of the isolated compounds (Gülşen *et al*. 2007).

#### HRMS spectrum

High-resolution mass spectrometry (HRMS) was employed to determine the molecular weight of the isolated compound (Parvez *et al*. 2021). The presence of a molecular ion peak was confirmed based on the data obtained from the HRMS analysis. The molecular weight of the isolated compound was determined using an HRMS Model Name: Waters-Xevo G2-XS-QToF mass spectrometer.

#### Antibacterial activity

Antibacterial activities of the tested sample were evaluated using well diffusion method on *Blood Agar*. The bacterial strains *H*. *pylori* were used as references for the antibacterial assay (Balouiri *et al*. 2016; Gonelimali *et al*. 2018; Wylie *et al*. 2022). *Blood Agar* plates were inoculated with bacterial strain under aseptic conditions was spread plated by glass L-rod with 100 μL grown culture. Then, squire (10 mm) compound coated thin film were attached on each plate, after that followed by incubation at 37°C for 24 h. After the incubation period, the zone of inhibition was measured and reported in millimeters (mm). The extracts were dissolved in distilled water and Chloramphenicol was used as control (Balouiri *et al*. 2016; Gonelimali *et al*. 2018; Wylie *et al*. 2022).

## RESULTS AND DISCUSSION

### Pharmacognostical Evaluation

The moisture content in mature stems and leaves was found to be 10.32% and 14.32%, respectively, based on loss on drying. These findings led us to conclude that this particular plant contains some minerals because the matured stem’s ash value was 4.4% and the stem and leaf’s ash value was 8.27%. For the standardisation of herbal medication, each of these factors is crucial.

### Qualitative Phytochemical Screening

According to the phytochemical analysis, flavonoids were found to be abundant in all dry powder extracts and highly abundant in fresh plant extracts. Phenols were present in most fresh plant part extracts, except for water extracts (MS, SL), while they were present in all dry powder extracts. Sterols were predominantly present in extracts from both fresh and dry plant parts, except for HMS extract. Alkaloids were detected in all extracts, both from fresh and dry plant parts, except for MSS and SLS. Furthermore, the phytochemical screening revealed that the extracts of *Ipomoea* plants contain a wide range of secondary metabolites, including tannins, terpenoids, carbohydrates, saponins, and fats and oils. The detailed results can be found in Tables 4 and 5.

### Quantitative Phytochemical Analysis

#### Estimation of secondary metabolites

Total phenolic content (TPC) of various crude extracts is quantified in terms of gallic acid equivalents (GAE), Flavonoids with various plant extracts quantified in terms of Quercetin equivalents (QE), tannins quantified in terms of Tannic acid equivalents (TE) and carbohydrate quantified in terms of Glucose equivalents (GE). All the quantitative estimation was tabulated in Table 6.

Significant differences were observed among the various stem extracts under investigation. Notably, the ethanolic matured stem extract (EMS) displayed the highest TPC, registering at 330.1 mg of GAE per gram. Meanwhile, HLS extract showcased a slightly lower TPC of 317 mg GAE/g. In terms of Quercetin content, it was found that all extracts, except for the DSH extract, contained minimal amounts of this compound. Notably, the Ethanolic Stem Extract (ESL) stood out by exhibiting the highest Quercetin content among all extracts, measuring at 569.4 mg of QE per gram. It is noteworthy that the presence of tannins was exclusively identified in the EMS and DMP extracts, with tannin concentrations recorded at 163.0 mg and 170 mg of TE per gram, respectively. All other extracts lacked tannin content. Furthermore, the DME was distinguished by its elevated Carbohydrate content, measuring at 783.5 mg of GE per gram. These findings underscore the diverse chemical compositions present within the examined extracts.

**Figure 4:**
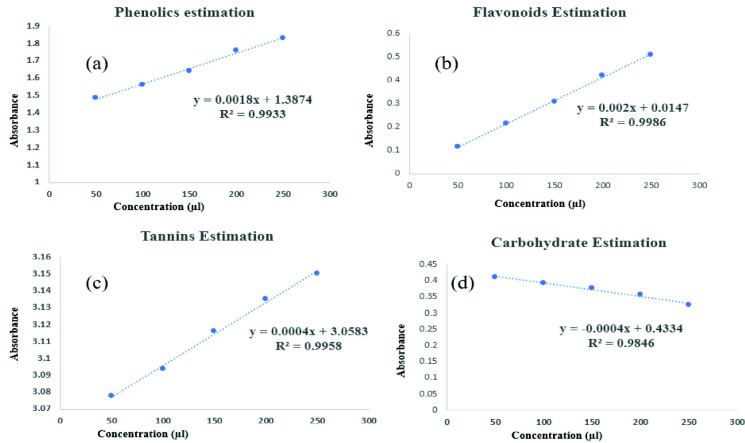
(a) Standard correlation graph of phenolics estimations; (b) standard correlation graph of flavonoids estimations; (c) standard correlation graph of tannins estimations; (d) standard correlation graph of carbohydrates estimations.

Fig. 4 shows the standard correlation graph of phenolics, flavonoids, tannins and carbohydrates estimations.

### Antibacterial Activity

Table 7 shows the antibacterial activity of *I. staphylina* extracts against *H. pylori*.

**Figure 5:**
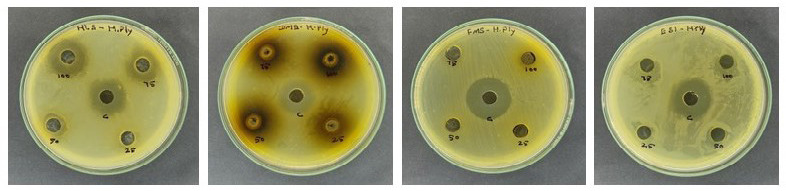
Antibacterial activity of *I. staphylina*.

In contrast to the control represented by chloramphenicol at a concentration of 100 μL, the HLS sample manifests a significant zone of inhibition, measuring 18 mm. Even at a lower concentration of 50 μL, a substantial inhibitory zone of 12 mm is observed. The DME extract, at 25 μL, exhibits a zone of inhibition measuring 14 mm, while at 100 μL, it demonstrates an expanded inhibitory zone of 17 mm. These findings underscore the efficacy of these extracts, particularly against Gram-negative bacteria such as *H. pylori*, hinting at a potential influence on cellular replication mechanisms. This antibacterial activity suggests promising avenues for further investigation and potential applications in combating bacterial infections (Fig. 5).

### Antioxidant Assay

**Figure 6:**
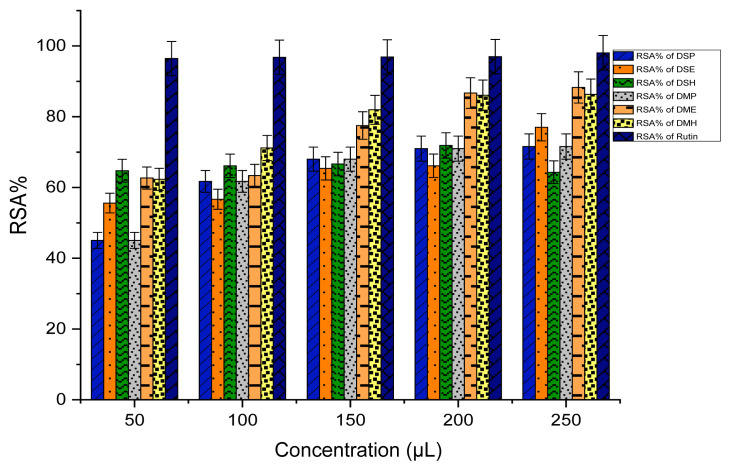
Antioxidant activity of (RSA%) dried extracts.

**Figure 7:**
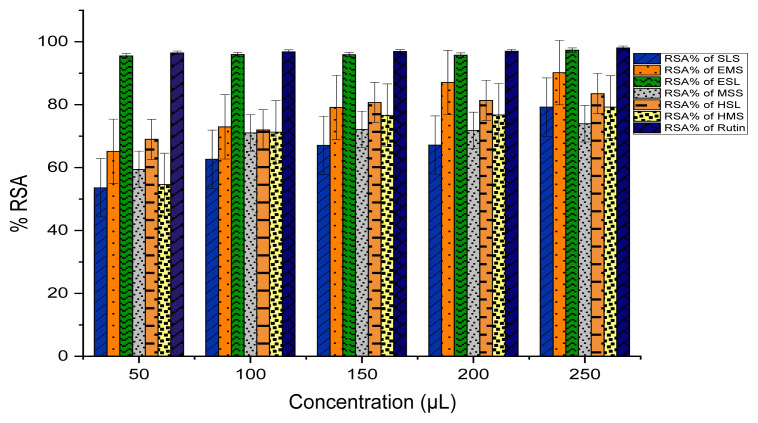
Antioxidant activity of (RSA%) fresh extracts.

DPPH stands as a stable free radical commonly employed in evaluating the capacity of extracts to function as radical scavengers or hydrogen donors, thereby assessing their antioxidant ability. The quantified outcomes of DPPH scavenging for extracts are succinctly presented, where Rutin serves as the standard compound, and these results are visually represented in Figs. 6 and 7. The efficacy of various extracts in attenuating the DPPH radical is conveyed through the metric of inhibitory percentage. Noteworthy is the pronounced DPPH radical inhibition showcased by the crude ESL, and EMS extracts, denoting an elevated level of activity. Moreover, the extracts DME and DMH exhibit a commendable yet moderate efficacy in comparison to the standard. All remaining extracts also exhibit favourable scavenging activity against the DPPH radical.

### HPTLC Analysis

**Figure 8:**
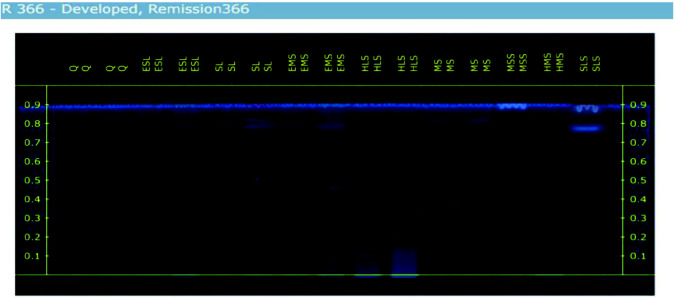
UV remission 366 of *I. staphylina* crude extracts.

**Figure 9:**
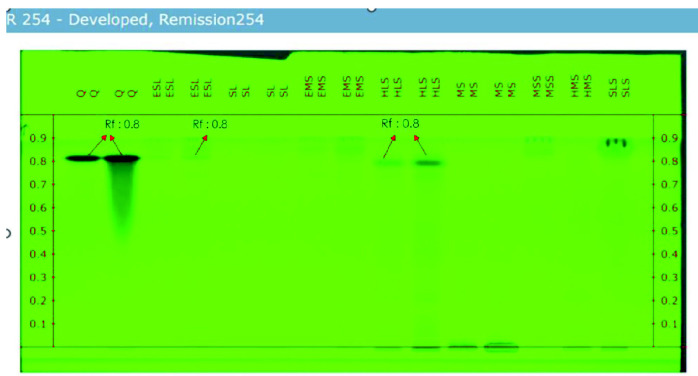
UV Remission 254 nm of *I. staphylina* crude extracts.

The HPTLC results indicate that among the tested extracts, EMS, HLS and EMS have the lowest concentration of Quercetin. Additionally, the preliminary phytochemical screening supports the strong presence of flavonoids in EMS, HLS and EMS. The R_f_ values of these three extracts match the reference R_f_ value (as shown in Table 8), confirming the presence of quercetin, a flavonoid. However, the HPTLC results reveal that quercetin is only present in trace amounts in all samples, except for SL and MS. Figs. 8 and 9 shows the HPTLC results of *I. staphylina*. Fig 8 shows UV remission 366 nm, and Fig 9 UV remission shows 254 nm.

Based on the HPTLC data, the R_f_ values of the standard quercetin (2 μL) were determined to be 0.787, 0.836 and 0.858, while for the 5 μL it showed 0.787, 0.826 and 0.858, respectively. Most of the samples exhibited very similar R_f_ values, indicating a comparable composition. Notably, the ESL (5 μL) sample displayed an end R_f_ value that precisely matched the end R_f_ value of quercetin (5 μL), suggesting that the ESL (5 μL) contains only a minimal quantity of quercetin. Furthermore, the beginning R_f_ values of HLS (2 μL and 5 μL) and HMS (2 μL) precisely matched the R_f_ value of quercetin, indicating that both samples contain only a small amount of quercetin. However, the results of the HPTLC analysis revealed that quercetin is present in trace amounts in all samples, except for SL and MS.

### FTIR Analysis

**Figure 10:**
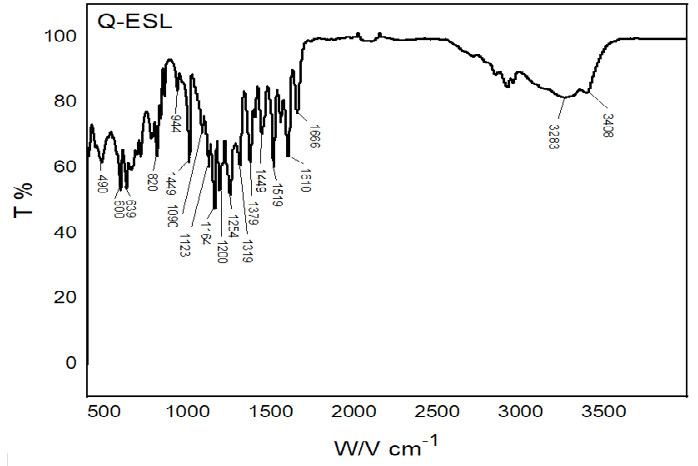
FTIR spectrum of Q-ESL and HLS.

Fig. 10 shows the FTIR spectrum of Q-ESL and HLS. The FTIR spectra results detected from the isolated compound (Q-ESL) confirm the OH phenolic stretch and hydroxyl stretch at 3408 cm^−1^ and 3283 cm^−1^, respectively. Stretching of aryl C=O at 1666 cm^−1^ C-C aromatic band shows at 1610 cm^−1^ and aromatic C=C vibration shows at 1519 cm^−1^, O-H bands of the phenolic group observe at 1379 cm^−1^, C-H bond starching observe at 1319 cm^−1^, C-O stretching shows at 1254 cm^−1^ and 1200 cm^−1^, aromatic C-H bending appeared at 944 cm^−1^, 820 cm^−1^, 639 cm^−1^, 600 cm^−1^ and 490 cm^−1^. The presented results of FTIR spectra conclude the conformation of the Quercetin functional groups.

### NMR Spectrum Analysis

NMR spectroscopy was employed to investigate the proton and carbon bonding in the isolated compound. The NMR analysis provided valuable insights into the structural characteristics of the compound, confirming the presence of quercetin. The obtained NMR results, which offer detailed information about the proton and carbon of Isolated compound is depicted in Figs. 9 and 10.

**Figure 11:**
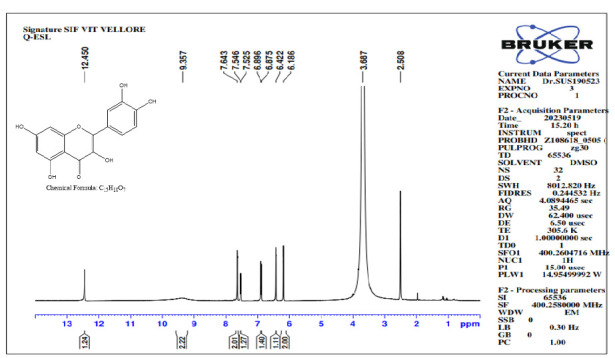
Proton NMR spectra of Q-ESL.

The ^1^H NMR spectrum of Quercetin (400MHz, DMSO-d_6_): In Fig. 11, the phenyl OH proton shows at the range of 12.450 ppm (s, H), OH proton at 9.357 ppm (s, 2H). The other two OH protons which have the same environment appeared at the range of 7.643 ppm (s, 2H). Aromatic protons appeared in the range of 7.546 ppm (d, *J* = 4.2Hz, 1H), 6.896 ppm (d, *J* = 4.2Hz, 1H), 6.422 ppm (s, H), 6.186 ppm (s, 2H). As per the result aromatic protons appeared in the 6.184 ppm–7.643 ppm region and the phenyl OH appeared in the 12.457 ppm and 9.381 ppm which strongly confirms the quercetin structure.

**Figure 12:**
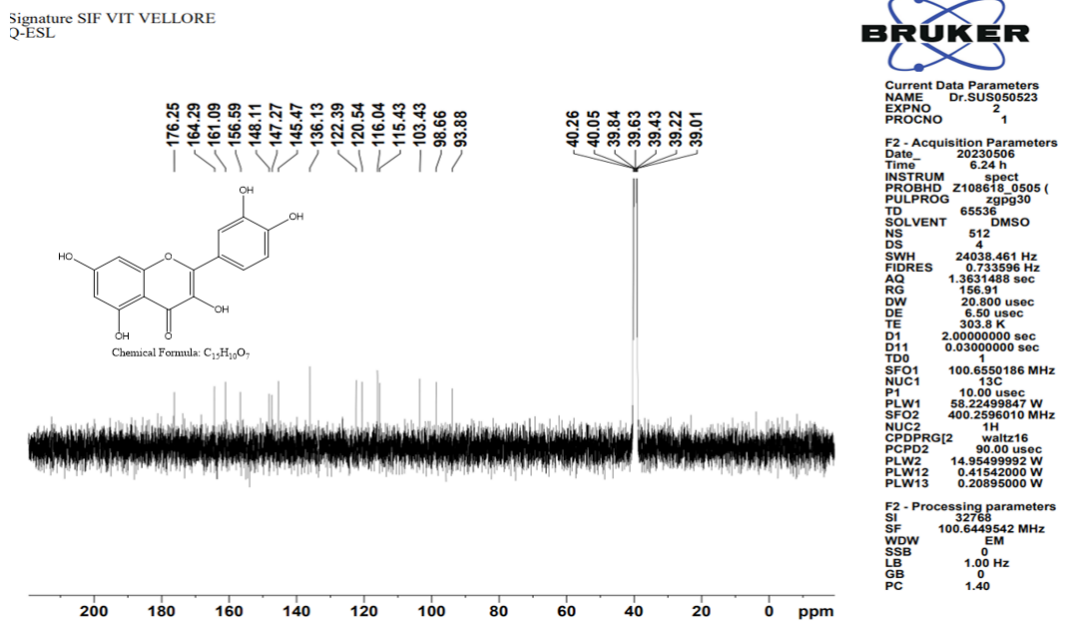
C^13^ Spectra of Q-ESL.

Fig.12 shows the ^13^C NMR spectrum of Quercetin. The ^13^C NMR spectrum of Quercetin (100 MHz, DMSO-d_6,_ δ) δ 176.25, 164.29, 161.01, 159.59, 148.11, 147.27, 145.47, 136.13, 122.39, 120.54, 116.04, 115.43, 103.43, 98.66, 93.88. The ^13^C spectra show that 176.25 ppm is a carbonyl carbon peak and the other attached carbons showed 164.29 ppm–156.59 ppm. The respective peaks of 148.11 ppm–122.39 ppm are carbons attached to the OH functional group. All the aromatic carbons appeared at 116.04 ppm–93.88 ppm. The advanced NMR spectrum results confirm the structure of Quercetin.

### HR-MS Spectroscopy

**Figure 13:**
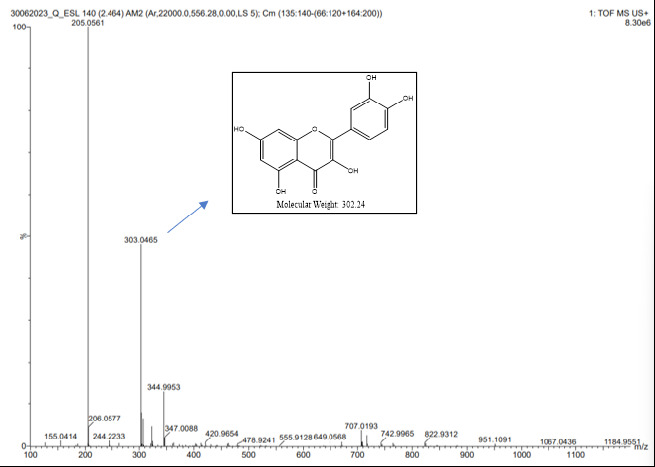
HRMS spectra of Q-ESL.

HR-MS spectroscopy was performed to conclude the molecular weight of the isolated compound. The spectrum confirms the molecular ion peak [M + H]^+^ at a range of 303.04 which is exactly similar to the Quercetin molecular weight (see Fig. 13).

The phytochemical screening of the extract derived from *I. staphylina* yielded compelling findings, highlighting its rich repertoire of secondary metabolites (Banerjee & Firdous 2020; Bashir *et al*. 2019; Harborne 1998). The comprehensive analysis unveiled the presence of various prominent classes of secondary metabolites, including phenols, alkaloids, carbohydrates, sterols, saponins, terpenoids, tannins, fats and oils, and flavonoids. These extensive phytochemical screenings, coupled with subsequent pharmacognostic investigations, accentuate the profound significance of *I. staphylina* as a valuable source of bioactive compounds.

Employing HPTLC, the presence of the flavonoid quercetin was effectively detected, consolidating its status as a constituent within the extract (Jain *et al*. 2021; Ved *et al*. 2022). The isolation of the pure bioactive component, quercetin, from the ethanolic extract of *I. staphylina* was accomplished using advanced instrumentation methodologies, facilitating its successful extraction and characterisation for further scientific exploration and potential applications.

The main objective of this research is to conduct a comprehensive comparison of crude extracts through pharmacognostical analysis and evaluate the antibacterial activity of matured stem extracts against *H. pylori* bacteria. Various parts of *I. staphylina* underwent rigorous pharmacognostical studies, leading to successful extraction with different solvents. Tables 1 and 2 present the outcomes of pharmacognostic evaluations and standardisation, showcasing the purity of *I. staphylina*. Qualitative and quantitative phytochemical analyses revealed the presence of crucial secondary metabolites, including phenols, alkaloids and flavonoids. Herbal drug quality was ensured through stringent tests assessing parameters like loss on drying and ash value, along with fluorescence analysis. Additionally, antioxidant potential was assessed using the DPPH assay, yielding promising results. HPTLC confirmed the presence of quercetin, which was isolated via column chromatography and further characterised using advanced techniques such as NMR, FTIR and HRMS (Davies & Johnson 2007; Meena & Patni 2008; Reid & Sarker 2012).

The primary objective of this research is to scrutinise the antibacterial efficacy against *H. pylori*, the causative agent of stomach ulcers. The focus is to substantiate, through scientific means, the traditional utilisation of *staphylina* for addressing stomach disorders and ulcers. Antibacterial assays reveal noteworthy effectiveness in HLS and DME ethanolic extracts derived from matured stems, as evidenced by substantial zones of inhibition against *H. pylori*. Concurrently, these extracts showcase elevated levels of secondary metabolites, with a pivotal bioactive compound isolated from the ESL extract. These secondary metabolites have the ability to disrupt the membrane of the test pathogens thereby leading to the apoptosis of the test pathogens (Baazeem *et al*. 2021; Odiba *et al*. 2014). These findings underscore the potential application of *I. staphylina* in treating stomach ulcers induced by *H. pylori*. Furthermore, this research provides scientific validation for the traditional use of *I. staphylina* in Dharmapuri for stomach ulcer management.

## CONCLUSION

In conclusion, this study has aimed to evaluate the antibacterial efficacy against *H. pylori*, the causative agent of stomach ulcers, with a specific focus on substantiating the traditional use of *staphylina* in addressing stomach disorders. The ethanolic extracts from matured stem extract demonstrated significant antibacterial activity, as indicated by substantial zones of inhibition against *H. pylori*. Moreover, these extracts exhibited heightened levels of secondary metabolites, including a crucial bioactive compound isolated from the *I. staphylina* plant extract. The identified secondary metabolites possess the capacity to disrupt the pathogen’s membrane, leading to the apoptosis of *H. pylori*. These findings emphasise the potential therapeutic application of *I. staphylina* in managing stomach ulcers induced by *H. pylori*. Additionally, the research contributes scientific validation for the traditional use of *I. staphylina* in Dharmapuri for the management of stomach ulcers, bridging traditional knowledge with empirical evidence.

## Figures and Tables

**Table 1:** Pharmacognostic evaluation *of I. staphylina* powder.

S. No	Parameters	Matured stem (%)	Stem and leaf (%)
1	Loss on drying	10.32	14.32
2	Ash value	4.40	8.27
3	Water soluble ash	-	2.60
4	Acid insoluble ash	-	-

Fluorescence studies employ estimations of fluorescence intensity as well as identify any fluorescent phytochemical compounds present in the plant.

**Table 2:** Fluorescence analysis of matured stem powder of *I. staphylina*.

S.No	Solvents	Visible	Long 365 nm	Short 254 nm
1	Powder	Brown	Brown	Light green
2	Water	Brown	Brown	Light green
3	Methanol	Brown	Brown	Light green
4	Ethanol	Brown	Brown	Light green
5	Ethyl acetate	Brown	Green	Light green
6	Petroleum ether	Brown	Light green	Light green
7	CHCl_3_	Brown	Green	Green
8	10%NaOH	Dark brown	Brownish green	Brownish green
9	10%KOH	Dark brown	Brownish green	Brownish green
10	HCl (4N)	Brown	Brown	Light green
11	H_2_SO_4_ (4N)	Brown	Brown	Light green
12	HNO_3_ (4N)	Dark brown	Green	Green
13	Acetic acid (4N)	Brown	Brown	Light green

**Table 3:** Fluorescence analysis of stem and leaf powders of *I. staphylina*.

S.No	Solvents	Visible	Long 365 nm	Short 254 nm
1	Powder	Brown	Brown	Light green
2	Water	Brown	Brown	Green
3	Methanol	Green	Dark green	Green
4	Ethanol	Light green	Green	Light green
5	Ethyl acetate	Light green	Green	Light green
6	Petroleum ether	Brown	Light green	Light green
7	CHCl_3_	Brown	Light green	Green
8	10%NaOH	Dark brown	Light green	Light green
9	10%KOH	Dark brown	Light green	Light green
10	HCl (4N)	Brown	Light green	Light green
11	H_2_SO_4_ (4N)	Brown	Light green	Light green
12	HNO_3_ (4N)	Brown	Green	Light green
13	Acetic acid (4N)	Brown	Light green	Green

**Table 4:** Fresh plant parts phytochemical screening report.

Secondary metabolites	Name of the test	MS	SL	MSS	SLS	EMS	ESL	HMS	HLS
Phenols	Neutral FeCl_3_ test	−	−	++	+	+	+	+	+
Flavonoids	Alkaline reagent test	+++	+++	+	+	+++	++	+++	+++
Alkaloids	Mayers test	+	++	−	−	++	+	+	+
Carbohydrates	Benedict’s test	−	−	−	−	−	++	−	+
Sterols	Salkowski test	+	+	+	+	+	+	−	+
Proteins	Biuret test	−	−	−	−	−	−	−	−
Saponins	Foam test	+++	+++	−	−	−	−	+	++
Terpenoids		+	+	+	+	+	+	+	+
Tannins		−	−	−	+	−	+	−	−
Fat and oils		−	−	++	++	++	++	−	−

*Note*: − Negative, + Present, ++ Moderate, +++ Strongly present. MS = fresh matured stem water extract; SL = fresh stem and leaf water extract; SLS = fresh stem and leaf pet-ether extract; MSS = fresh matured stem pet-ether extract; EMS = fresh matured stem ethanol extract; ESL = fresh stem and leaf ethanol extract; HLS = fresh stem and leaf hydroalcoholic extract; HMS, fresh matured stem hydroalcoholic extract

**Table 5:** Dried plant parts phytochemical screening report.

Secondary metabolites	Name of the test	DSP	DMP	DSE	DME	DSH	DMH
Phenols	Neutral FeCl_3_ test	+	+	−	−	+	+
	Gela	++	++	+	+	−	+
Flavonoids	Alkaline reagent test	+	+	+	+	+	+
Alkaloids	Mayers test	++	++	+	++	+	+
Carbohydrates	Benedict’s test	−	−	+	++	++	+++
Sterols	Salkowski test	+	+++	++	+++	++	++
Proteins	Biuret test	−	−	−	−	−	−
Saponins	Foam test	−	−	−	+	++	++
Terpenoids	Copper acetate test	+	+	+	+	++	++
Tannins		−	−	+	+	+	+
Fat and oils		+	+	+	+	−	−

*Notes*: − Negative, + Present, ++ Moderate, +++ Strongly present. DMP = dried matured stem pet-ether extract; DSP = dried stem and leaf pet-ether extract; DSE = dried stem and leaf ethanol extract; DME = dried matured stem ethanol extract; DSH = dried stem and leaf hydroalcoholic extract; DMH = dried matured stem hydroalcoholic extract.

**Table 6:** Quantitative estimation of plant extracts.

S. No	Sample code	Total phenolics (mg/g)	Total flavonoids (mg/g)	Total tannins (mg/g)	Total carbohydrates (mg/g)
1	SLS	113.2	242.3	-	-
2	ESL	226.3	569.4	-	401.9
3	HLS	317.5	256.2	-	325.6
4	MSS	-	196.0	-	-
5	EMS	330.1	128.1	163.0	-
6	HMS	-	43.5	-	-
7	DSP	-	256.6	-	-
8	DSE	-	282.7	-	87.7
9	DSH	110.4	-	-	670.4
10	DMP	28.5	497.6	170.7	-
11	DME	36.6	567.3	-	783.5
12	DMH	-	115.3	-	-

**Table 7:** Antibacterial activity results.

S.No	Sample name	*H*. *pylori* (Zone of inhibition (mm) Concentration (μL)			

		25 μL	50 μL	75 μL	100 μL
1	Chloramphenicol	16	15	20	21
2	*HLS*	-	12	15	18
3	*DME*	14	15	16	17
4	*EMS*	-	-	-	-
5	*ESL*	-	-	-	-

**Table 8:** HPTLC results.

S. No	Compound	No. of peaks	Start R_f_	Max R_f_	End R_f_	Area	Percentage (%)
1	Quercetin 2 μL	1	**0.787**	**0.826**	**0.858**	0.02430	100
2	Quercetin 5 μL	1	**0.787**	**0.833**	**0.854**	0.01799	100
3	ESL 2 μL	1	0.804	0.828	**0.854**	0.00101	100
4	ESL 5 μL	1	0.813	0.828	**0.854**		100
5	SL 2 μL	-	-	-	-	-	-
6	SL 5 μL	1	0.822	0.836	0.851	0.00022	100
7	EMS 2 μL	-	-	-	-	-	-
8	EMS 5 μL	2	0.796	0.813	0.822	0.00015	38.06
			0.825	0.849	0.857	0.00025	61.94
9	HLS 2 μL	1	**0.787**	0.806	0.833	0.00600	100
10	HLS 5 μL	1	**0.787**	0.807	0.860	0.00858	100
11	MS 2 μL	-	-	-	-	-	-
12	MS 5 μL	-	-	-	-	-	-
13	MSS 5 μL	1	0.819	0.844	0.861	0.00142	100
14	HMS 5 μL	1	**0.787**	0.806	0.824	0.00052	100
15	SLS 5 μL	1	0.813	0.838	0.851	0.00102	100

*Notes:* ESL = fresh stem and leaf ethanol extract; SL = fresh stem and leaf water extract; EMS = fresh matured stem ethanol extract; HLS = fresh stem and leaf hydroalcoholic extract; MS = fresh matured stem water extract; MSS = fresh matured stem pet-ether extract; HMS = fresh matured stem hydroalcoholic extract; SLS = fresh stem and leaf pet-ether extract. The bold numbers represent the standard R_f_ values, while other bold numbers indicate matches with these standard R_f_ values.
